# Dermatological conditions during TNF-α-blocking therapy in patients with rheumatoid arthritis: a prospective study

**DOI:** 10.1186/ar1724

**Published:** 2005-04-04

**Authors:** Marcel Flendrie, Wynand HPM Vissers, Marjonne CW Creemers, Elke MGJ de Jong, Peter CM van de Kerkhof, Piet LCM van Riel

**Affiliations:** 1 Department of Rheumatology, Radboud University Nijmegen Medical Centre, Nijmegen, the Netherlands; 2 Department of Dermatology, Radboud University Nijmegen Medical Centre, Nijmegen, the Netherlands

## Abstract

Various dermatological conditions have been reported during tumor necrosis factor (TNF)-α-blocking therapy, but until now no prospective studies have been focused on this aspect. The present study was set up to investigate the number and nature of clinically important dermatological conditions during TNF-α-blocking therapy in patients with rheumatoid arthritis (RA). RA patients starting on TNF-α-blocking therapy were prospectively followed up. The numbers and natures of dermatological events giving rise to a dermatological consultation were recorded. The patients with a dermatological event were compared with a group of prospectively followed up RA control patients, naive to TNF-α-blocking therapy and matched for follow-up period. 289 RA patients started TNF-α-blocking therapy. 128 dermatological events were recorded in 72 patients (25%) during 911 patient-years of follow-up. TNF-α-blocking therapy was stopped in 19 (26%) of these 72 patients because of the dermatological event. More of the RA patients given TNF-α-blocking therapy (25%) than of the anti-TNF-α-naive patients (13%) visited a dermatologist during follow-up (*P *< 0.0005). Events were recorded more often during active treatment (0.16 events per patient-year) than during the period of withdrawal of TNF-α-blocking therapy (0.09 events per patient-year, *P *< 0.0005). The events recorded most frequently were skin infections (*n *= 33), eczema (*n *= 20), and drug-related eruptions (*n *= 15). Other events with a possible relation to TNF-α-blocking therapy included vasculitis, psoriasis, drug-induced systemic lupus erythematosus, dermatomyositis, and a lymphomatoid-papulosis-like eruption. This study is the first large prospective study focusing on dermatological conditions during TNF-α-blocking therapy. It shows that dermatological conditions are a significant and clinically important problem in RA patients receiving TNF-α-blocking therapy.

## Introduction

The introduction of biological agents such as TNF-α-blocking agents has dramatically changed the therapeutic approach to rheumatic diseases in recent years. TNF-α-blocking therapy has had a remarkable effect on disease activity in an increasing number of rheumatic diseases, including rheumatoid arthritis (RA) [1-3], juvenile idiopathic arthritis [4], ankylosing spondylitis [5,6], and psoriatic arthritis [7]. At present, two monoclonal anti-TNF-α antibodies (infliximab and adalimumab) and one soluble p75 TNF-α receptor (etanercept) are being used in rheumatological practice.

Various skin conditions have been reported in clinical trials, including urticaria, rash, and stomatitis (during infliximab therapy) [8]; rash and injection-site reactions (during adalimumab therapy) [3,9]; and injection-site reactions (during etanercept therapy) [2].

However, clinical trials are not designed to provide information about the occurrence of rare adverse events associated with TNF-α-blocking therapy. More severe cutaneous reactions, such as erythema multiforme, discoid and subacute cutaneous lupus erythematosus, atopic dermatitis, necrotizing vasculitis, and bullous skin lesions, have been reported, mostly as single-case observations [10-15]. Larger observational studies such as biological registries are needed to provide information on the nature and number of such dermatological adverse events during TNF-α-blocking therapy.

The aim of this study was to investigate whether dermatological conditions after TNF-α-blocking therapy are a significant and clinically important problem in RA patients receiving TNF-α-blocking therapy.

## Materials and methods

### Study design

In a prospective cohort study, all consecutive patients with a diagnosis of RA according to the criteria of the American Rheumatism Association [16] who were starting on TNF-α-blocking therapy at the Department Of Rheumatology of the Radboud University Nijmegen Medical Centre were followed as part of a Biological Registry [17]. Approval was obtained by the hospital's ethics committee.

Patients were required to meet the criteria set out in the Dutch guidelines for biological therapies: a moderate to high disease activity score (DAS) based on 28 joints (DAS28 ≥ 3.2), and failure or intolerability of at least two disease-modifying antirheumatic drugs (DMARDs), including methotrexate, in adequate dosage regimens. Besides therapy with registrated TNF-α-blocking agents – infliximab, etanercept, and adalimumab – some patients were treated in clinical trials with lenercept, a soluble p55 TNF-α-receptor [18].

The number and nature of dermatological conditions that led patients in this cohort to consult a dermatologist during follow-up were investigated. The RA patients treated with TNA-α-blocking agents who experienced dermatological events was compared with a control group of patients who had RA but had never had TNF-α-blocking therapy. The control patients were selected from the Nijmegen inception cohort, in which 500 RA patients have been followed since 1985 [19]. Each control was paired with a TNF-α-treated patient for duration and season of the follow-up period, within a 2-month window.

### Variables

Data collected at the start of TNF-α-blocking therapy were age, sex, duration of disease, presence or absence of rheumatoid factor (measured by ELISA; considered positive if results showed >10 IU/ml), antinuclear antibody (tested for by immunofluorescence on Hep-2 cells), number of DMARDs previously used, and start date of TNF-α-blocking therapy. Baseline information obtained included erythrocyte sedimentation rate (ESR), 28-joint counts for swelling and tenderness, and general wellbeing as indicated on a visual analogue scale, and the disease activity score (DAS28) was calculated [20].

Variables about which information was collected during TNF-α-blocking therapy were the use of concomitant DMARDs and prednisolone, dose and interval changes of TNF-α-blocking agents and, if appropriate, date and reason for discontinuation.

All patients who visited a dermatologist during follow-up were identified. Clinically important dermatological events were defined as any new manifestation or any exacerbation of pre-existing skin disease during follow-up. A standardized chart review form was used to record the following: start date of event, dermatological history, medication, morphological description, localization, histopathological and immunohistological information if available, working diagnosis, additional investigations, topical and systemic therapeutic actions, outcome of event, and any available information on rechallenge.

Drug-related eruptions were defined as skin reactions with a probable or definite relation to the use of TNF-α-blocking agents, based on a time relation with the administration of the agent, morphological pattern, and/or histological information. Drug-related eruptions were classified morphologically according to the criteria of Fitzpatrick and colleagues [21]. Events were also classified as major or minor, major events being any requiring hospitalization.

Patient-years of follow-up were calculated for total follow-up, time on active therapy, and time after discontinuation of therapy (time off therapy). The number of events per year of follow-up was calculated for each RA patient for total time of follow-up, time on active treatment, and time off treatment, if appropriate.

In the control group, the following baseline characteristics were collected: age, sex, disease duration, rheumatoid factor, antinuclear antibody, DAS28, the number of DMARDs previously used, and prednisolone use. All visits to a dermatologist during follow-up were identified. Events were not recorded in the control group.

### Statistical analyses

The baseline characteristics of RA patients on TNF-α-blocking therapy were compared according to whether or not the patients experienced dermatological events. The chi-square test was applied for dichotomous variables and Student's *t*-test was used for continuous variables. Nonparametric tests were applied when appropriate. The Wilcoxon signed rank test was used to compare the number of events per patient-year of follow-up in patients receiving and patients not receiving active TNF-α-blocking therapy. Univariate and multivariate logistic regression analyses were performed to identify possible predictive factors for the occurrence of a dermatological visit (independent variable, dichotomous) in RA patients on TNF-α-blocking therapy. Dependent variables tested were sex, age at diagnosis, rheumatoid factor, antinuclear antibody, disease duration, DAS28 at baseline, prior number of DMARDs, use of prednisolone, and duration of follow-up. Odds ratios (ORs) and 95% confidence intervals (95% CIs) were calculated.

The number of patients who visited a dermatologist was compared between RA patients on TNF-α-blocking therapy and controls, using the chi-square test. *P *values and ORs were calculated.

All tests were two-sided, with *P *< 0.05 considered statistically significant. Statistical analyses were performed using SPSS statistical software (v 12.0.1, SPSS Inc, USA).

## Results

### Patients

A total of 289 RA patients started TNF-α-blocking therapy between June 1994 and December 2003. Their baseline characteristics are shown in Table 1.

The median follow-up time was 2.3 years (range 0.02 to 9.6). The total follow-up time was 911 patient-years, with 627 patient-years representing active therapy. Seventy of the 289 RA patients (24%) received more than one TNF-α-blocking agent and 8 (3%) received more than two agents. Infliximab was administered to 167 patients, adalimumab to 108, etanercept to 78, and lenercept to 31.

Dermatological events were recorded in 72 of the 289 RA patients (25%) receiving TNF-α-blocking therapy and in 37 (13%) of the control group (*n *= 289). The odds ratio (OR) of TNF-α-blocking therapy for a dermatological referral was 2.26 (95%CI 1.46 to 3.50, *P *< 0.0005). In patients on TNF-α-blocking therapy fifty-six instances of dermatological conditions were recorded in 34 patients (47%) and included, among others, 10 drug reactions – while the patient was receiving gold (7), nonsteroidal anti-inflammatory drugs (2), or methotrexate (1) – 10 cases of eczema, 9 of mycosis, 3 of other infections, and 5 of chronic venous insufficiency.

### Predictive factors

In univariate analyses, duration of follow-up (OR 1.27, 95%CI 1.14 to 1.41, *P *< 0.0005) and of disease (OR 1.03, 95%CI 1.003 to 1.07, *P *< 0.05) were statistically significant predictive factors for a dermatological event. In a multivariate model, only duration of follow-up was a statistically significant predictive factor (OR 1.30, 95%CI 1.12 to 1.52, *P *< 0.001).

### Dermatological events

One hundred and twenty-eight dermatological events were recorded during follow-up in RA patients on TNF-α-blocking therapy (0.14 event per patient-year), as listed in Table 2. The event per patient-year ratio was 0.16 during active treatment and 0.10 off treatment (*P *< 0.001). The number of events recorded during or after treatment was 56 for adalimumab (0.12 event per patient-year), 49 for infliximab (0.14 per patient-year), 16 for etanercept (0.13 per patient-year), and 13 for lenercept (0.07 per patient-year). TNF-α-blocking therapy was permanently withdrawn because of dermatological events 21 times in 19 patients.

### Infections

Thirty-three infections were recorded in 27 patients, consisting of 20 fungal, 11 bacterial, and 2 viral infections (see Table 3). Two patients had had a previous episode of dermatomycosis. None of the patients required hospitalization. One patient, who temporarily discontinued adalimumab monotherapy twice because of elective surgery, developed a bacterial superinfection of pre-existing eczema after every restart.

### Eczema

Eczema was diagnosed 20 times in 19 patients and appeared in various morphological patterns. Most events were described as erythematosquamous (*n *= 8) or erythematous (*n *= 3) lesions or plaques, localized on hands and feet (*n *= 3), arms and legs (*n *= 5), face (*n *= 1), neck (*n *= 1), and buttocks (*n *= 1). A vesicular rash on hands and feet was described five times. A papular rash was described in three cases, with localization around the eyes, on the back, and once on the back and lower legs. Diagnoses comprised dyshidrotic (*n *= 5), contact (*n *= 4), nummular (*n *= 1), atopic (*n *= 1), papular (*n *= 1), and nonspecific eczema (*n *= 8). Two patients had a prior history of dyshidrotic eczema.

Biopsies were performed in five events. Histology showed dermatitis and spongiosis in all cases, with high dermal perivascular infiltration in three. One biopsy also showed mild psoriasiform acanthosis and another showed additional keratinocyte necrosis.

Three patients stopped TNF-α-blocking therapy because of the dermatological event, after which the lesions resolved. Hospitalization was necessary for treatment of eczema in one patient. In another patient the eczematous lesions recurred after adalimumab therapy was restarted. Adalimumab was continued and topical steroids were applied with good effect. TNF-α-blocking therapy had already been stopped in 4 patients before the onset of eczema and was continued in 13 patients, of whom 7 had persisting or recurring lesions. Therapy consisted mostly of topical corticosteroids.

### Drug-related eruptions

Drug-related eruptions occurred frequently during the first 5 months of TNF-α-blocking therapy and were caused by all four TNF-α-blocking agents (see Table 4). In two cases, a generalized drug-related eruption followed subcutaneous injection of etanercept. In two cases, the eruption developed during infusion (patients numbers 8 and 11, Table 4). In the other cases the time of onset ranged between 2 and 57 days after the most recent infusion.

Most drug-related eruptions consisted of a combination of morphological patterns, including exanthema, urticarial eruptions, lichenoid skin lesions, and purpura. In four patients, an eczematous drug-related eruption was seen. Classification as drug-related eruption was based on a time relation with administration of the TNF-α-blocking agent, the morphological pattern, and/or histological information. Two patients had experienced a previous drug-induced eruption (1 dermatitis in response to gold, 1 dermatitis after indomethacin).

The histological findings were compatible with the diagnosis in all cases. Perivascular infiltrations – predominantly lymphocytic – epidermal exocytosis, and hyperorthokeratosis were described. Interface dermatitis was described in three instances. One biopsy revealed focal infiltrations with marked vascular and endothelial proliferation.

**Figure 1:**
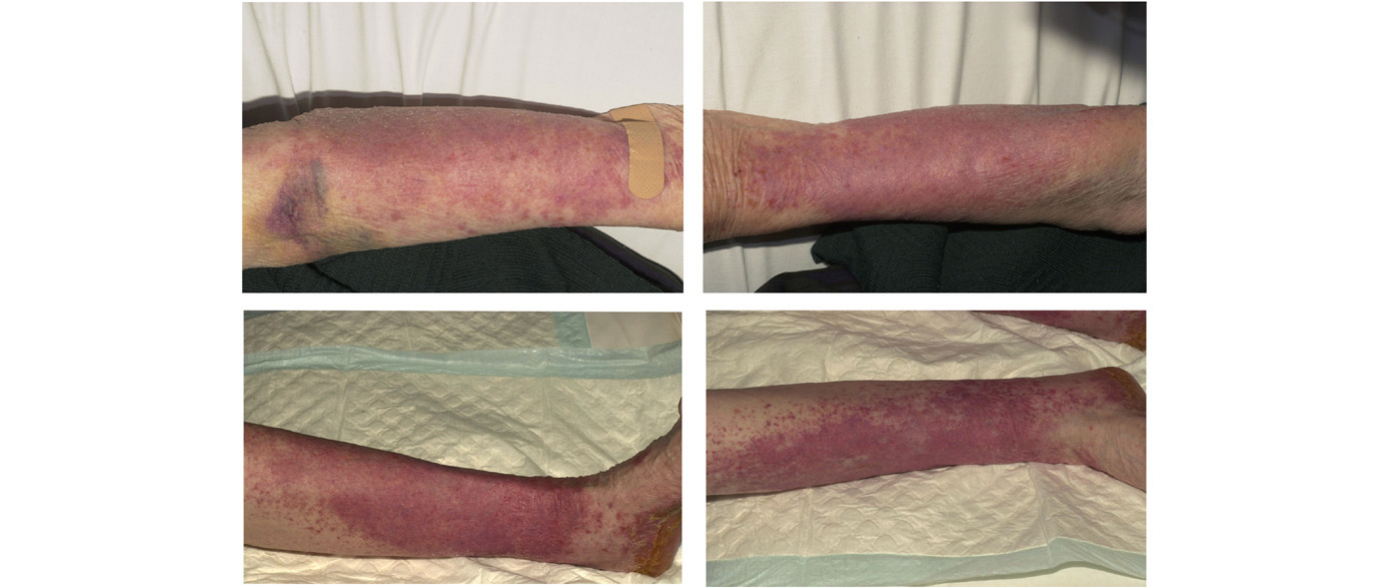
Eczematous drug-related eruption a patient with rheumatoid arthritis after infliximab therapy: Eczematous eruptions on the left arm (top left) and right arm (top right) and erythematous eruptions with purpura on the left leg (bottom left) and right leg (bottom right).

Seven patients stopped and 8 patients continued therapy; 6 of them had a positive rechallenge and recurring lesions. One major event was recorded: an RA patient was hospitalized for an extensive eczematous eruption with urticaria on arms and legs (Fig. 1, and Patient no. 6 in Table 4). Treatment consisted mostly of topical application of corticosteroids and sometimes of systemic antihistamines.

### Tumors and actinic keratosis

Events of skin malignancies were recorded five times, in four patients. One RA patient developed three basal cell carcinomas simultaneously on her left arm, right nostril, and right eyelid after 2.7 years of adalimumab therapy, which was subsequently stopped. One 74-year-old RA patient developed Bowen's disease on his right hand 2 years after adalimumab therapy had been stopped. The same patient later developed a squamous cell carcinoma on the left earlobe after the start of etanercept therapy. Other skin malignancies recorded were a squamous cell carcinoma (earlobe) after 1.5 months of adalimumab therapy and a low-grade basalioma (Pinkus epithelioma) on the leg after 6 months of adalimumab therapy. In all cases, histology confirmed the diagnosis and therapy consisted of excision. No recurrences were seen.

Actinic keratosis was recorded in five patients (three receiving adalimumab, one infliximab, and one lenercept). Excision or cryotherapy was successful in four. One patient had recurring actinic lesions on the scalp.

Benign tumors were recorded seven times during TNF-α-blocking therapy. One patient experienced an increased growth of a facial telangiectatic nevus, present since childhood, 2 months after starting etanercept therapy. Seborrheic keratosis (*n *= 3), oral hyperkeratosis (*n *= 1), histiocytoma (*n *= 1), and fibroma (*n *= 1) were also recorded.

### Vasculitis

Vasculitis was recorded five times: four during and one after cessation of TNF-α-blocking therapy. The diagnosis was confirmed by biopsy in four cases. One patient developed a superficial necrotizing leukocytoclastic vasculitis with ulceration after 7 months of infliximab therapy, with complete recovery after discontinuation of infliximab. One patient developed a papular erythema in the groins after 5 years of adalimumab therapy. Histological examination was compatible with vasculitis with infiltration of mononuclear cells and presence of eosinophilic granulocytes. One patient developed a purpuric vasculitis on the legs after 1.5 months of lenercept therapy, improving spontaneously despite continuation of lenercept. One patient developed isolated digital vasculitis on his toes after one year of adalimumab therapy, which was continued. The lesions persisted. No biopsy was performed. One patient developed a generalized urticarial exanthema after therapy with etanercept 2 years earlier. Current therapy consisted of hydroxychloroquine and prednisolone. Histology showed a mild leukocytoclastic vasculitis.

### Ulcers

The nine events with ulcers included four pressure ulcers, two ulcers due to dependency edema, one traumatic ulcer, one ulcer secondary to an unguis incarnatus, and one ulcer without further specification. Biopsies were taken in two patients, but no signs of vasculitis were found. A patient had a pressure ulcer with secondary infection and a fistula on his ankle, which contained osteosynthetic material. The patient was admitted to the hospital for intravenous antibiotic therapy and infliximab was stopped for several months. After recovery, the patient restarted infliximab without recurrence of his skin problems. TNF-α-blocking therapy was continued in the other eight patients, and in four of these the ulcers recovered; follow-up was missing in the other four.

### Stasis dermatitis, edema, varices and chronic venous insufficiency

In 10 patients, a dermatological consultation was recorded for stasis dermatitis (*n *= 3), edema (*n *= 3), varices (*n *= 2), or chronic venous insufficiency (*n *= 2). In one patient with extensive varices, infliximab therapy was stopped temporarily because of a complicating thrombophlebitis. One patient had edema of both legs of unknown cause, with livid discoloration and induration. One patient had lymphedema secondary to RA. All other events were considered to be related to comorbidity, other than RA.

### Psoriasis and psoriasiform eruptions

Psoriatic or psoriasiform eruptions were recorded in three RA patients. One developed a vesiculopustular erythematosquamous rash on hands and feet after 9 months of adalimumab therapy. Histology showed a mixed psoriasiform and spongiotic dermatitis. A second RA patient developed psoriasis guttata-like eruptions on her lower legs after 4 years of therapy with adalimumab. The lesions diminished after adalimumab was withdrawn. A third patient developed a psoriasiform eruption on arms and legs after 16 months of adalimumab therapy. Histology obtained in the latter two patients was consistent with psoriasis.

### Other dermatological conditions

Other dermatological conditions that occurred during or after TNF-α-blocking therapy included, among others, dermatomyositis (1), drug-induced systemic lupus erythematosus (1), and lymphomatoid papulosis-like eruption (1). Details are shown in Table 5.

One RA patient developed a macular rash on the inner sides of the upper arms and legs after 2.5 months of lenercept monotherapy. A skin biopsy showed a nonspecific chronic dermatitis. A soft-tissue biopsy, including skin, fascia, and muscle, showed fascial and muscular infiltration, consistent with dermatomyositis.

One RA patient developed a drug-induced systemic lupus erythematosus after 20 months of infliximab therapy in combination with methotrexate, consisting of discoid lupus erythematosus lesions on her hands and scalp, aphthous lesions, conversion to antinuclear antibody positivity, and a positive anti-double stranded-DNA (titer 60 U/L). The skin lesions flared within one week after infusion and disappeared after discontinuation of infliximab.

A third RA patient developed macular erythematosquamous lesions on her lower arms, upper legs and trunk after 2.6 months of adalimumab monotherapy. Histology showed a dermal infiltration with CD30-positive atypical T cells. Although the lesions appeared to be lymphomatoid papulosis, they completely disappeared within 6 weeks. Adalimumab was not stopped. This patient developed a large-cell anaplastic non-Hodgkin lymphoma 2 years later.

## Discussion

The present study is the first large prospective study focusing on dermatological conditions in RA patients on TNF-α-blocking therapy. Of the patients studied, 25% needed a dermatological consultation, compared with 13% in a RA control group, naive to TNF-α-blocking therapy. The number of dermatological events per patient-year was significantly higher during treatment than after treatment with TNF-α-blocking therapy. Dermatological events led to withdrawal of TNF-α-blocking therapy in 19 patients of 72 patients (26%). The events recorded most frequently were skin infections, eczema, and drug-related eruptions. Some other interesting events were recorded, such as psoriasis, drug-induced systemic lupus erythematosus, dermatomyositis, and a lymphomatoid-papulosis like eruption.

RA is known to be associated with dermatological conditions such as vasculitis, nodulosis, palmar erythema, and bullous pemphigoid, among others [22,23]. At present, information on the incidence and prevalence of dermatological conditions in RA mainly originates from cross-sectional or retrospective studies [24-26]. Few prospective studies have been conducted focusing on specific conditions affecting the skin [27,28].

In establishing a relation between the use of a drug and the occurrence of dermatological conditions, various factors must be considered. Information on clinical and histological patterns, time and dose relation, dechallenge and rechallenge, and analogy with previously reported cases can provide support in assessing the plausibility of such a relation [29]. The underlying disease and concomitant medication also need careful consideration, as they can provide alternative explanations.

In this study the largest group of dermatological events consisted of skin infections, mostly fungal infections and folliculitis. The use of TNF-α-blocking therapy has raised concerns regarding an increased susceptibility to infections, as TNF-α plays an important role in host-defence mechanisms [30]. An increased incidence of tuberculosis has been described [31], as well as a growing number of serious infections with fungal, mycobacterial, and intracellular bacterial pathogens [32-34]. Infections of the skin have not been the subject of report in clinical trials and observational studies with TNF-α-blocking therapy. Cases of severe necrotizing fasciitis have been described [35,36].

Skin infections have been reported frequently in the normal population and especially in RA patients [24-26]. Host-defence impairments resulting from the underlying disease might play a role in an increased susceptibility to skin infections in RA patients, as well as the use of corticosteroids and DMARDs such as methotrexate [28,37], which were recorded frequently in the present study (see Table 2). They could provide an alternative explanation for the occurrence of skin infections. However, most infections occurred during active treatment with TNF-α-blocking therapy, a finding that could suggest at least a relative contribution to an increased vulnerability to skin infections in the study population. In one patient, a bacterial superinfection of eczema occurred twice immediately after restart of adalimumab, showing a clear time relation.

For the description of the recorded drug-related eruptions, a clinico-morphological classification was chosen [21]. Four eruptions with a time relation and clinically or histological distinct drug-induced patterns also showed an eczematous appearance, both clinically and histologically. This is an unusual presentation for a drug-induced eruption and warrants further investigation.

Two drug-related eruptions occurred during infusion with infliximab or adalimumab, whereas all the others occurred after infusion. This will most likely not reflect the true ratio between acute and delayed reactions involving the skin, since acute reactions with skin involvement occur in 4% of the infusions and are usually treated by the rheumatologist without dermatological consultation [38].

Eczema was reported frequently in this study, even with various dermatitis conditions, such as xerosis cutis, stasis eczema, and seborrheic eczema, classified as separate entities. Previous studies have reported RA, in which Th1 (T helper cell type 1) immune responses dominate, to be negatively associated with Th2-cell-mediated atopic disorders, such as eczema [39-41], although a similar incidence of eczema in RA and non-RA patients has also been reported [42]. TNF-α-blocking therapy down-regulates Th1 immune responses [43], which might induce a shift of the Th1/Th2 balance towards Th2-dominated immune responses and which might promote an increased susceptibility to atopic disorders, such as eczema.

Although the time between the initiation of TNF-α-blocking therapy and the onset of dermatological conditions varied, a probable relation was seen in various events. These included, besides drug-related eruptions, events of cutaneous vasculitis, drug-induced systemic lupus erythematosus, dermatomyositis, and a lymphomatoid papulosis-like eruption.

An association between the use of TNF-α-blocking therapy and the induction of systemic lupus erythematosus and discoid lupus erythematosus is strongly suggested by the number of cases that have been published [10,11,13,44-46]. One case of discoid lupus erythematosus has been described on both etanercept and infliximab in the same RA patient [47].

Analogy with previous reports is also present for cutaneous vasculitis [13,47-49], although it is a known extra-articular manifestation of RA [22,23]. In the first case described, a probable relation with infliximab was present, based on the time relation and positive dechallenge. The other cases described were considered possibly related (Results section, Vaculitis, cases 2 and 3) and unlikely (cases 4 and 5). Almost all reported ulcers were considered secondary to other causes, as described.

Dermatomyositis has been reported previously, although the patient affected in that case had a different presentation, with raised creatinine phosphokinase, muscle atrophy, mechanic's hands, and vasculitis [17].

Another interesting finding was the occurrence of psoriasiform eruptions in three patients on TNF-α-blocking therapy. This observation is particularly interesting, since etanercept has received and infliximab is close to receiving FDA approval for treatment of psoriasis, after remarkable efficacy results in clinical trials [7,50,51]. The occurrence of guttate psoriasis has been reported after initiation of etanercept therapy for psoriasis in a placebo-controlled trial [51]. Another case report described the occurrence of psoriasiform eruptions with histologically a lichenoid dermatitis pattern in a patient with Crohn's disease [52].

An exacerbation of psoriasis was also seen in a patient with psoriatic arthritis receiving infliximab therapy. An additional analysis showed that 28 patients with various non-RA rheumatic diseases, including 12 juvenile idiopathic arthritis, 6 psoriatic arthritis, and 3 ankylosing spondylitis, had been treated with TNF-α-blocking therapy in the study centre. Five patients (18%) had visited a dermatologist for a dermatological condition during or after TNF-α-blocking therapy. The events included a drug-related eruption, eczema, and a facial mycosis in three patients with juvenile idiopathic arthritis and a superficial spreading melanoma in a patient with ankylosing spondylitis. This indicates that the occurrence of dermatological events during TNF-α-blocking therapy is not restricted to RA patients.

In the present study the control patients were matched for sartdate and duration of follow-up period in order to control for time-related effects. A statistically significant relation between the use of TNF-α-blocking therapy and the occurrence of dermatological visits was shown. The two groups studied differed for most baseline characteristics. These differences result from the indication for TNF-α-blocking agents, which were reserved for patients who fulfilled criteria for active disease and DMARD failure (see methods section; study design), had a longer disease duration, and whose disease was perhaps more refractory.

However, it is considered unlikely that these factors influenced the relation between the use of TNF-α-blocking therapy and dermatological visits. In a multivariate regression model, no baseline characteristic showed a predictive value for the occurrence of a dermatological event in RA patients on TNF-α-blocking therapy. Also, a statistically significantly higher number of dermatological events was recorded during active treatment with TNF-α-blocking therapy than after the therapy had been stopped.

## Conclusion

This is the first prospective study showing a relation between TNF-α-blocking therapy and the occurrence of dermatological conditions. Future prospective studies are needed to investigate the incidence and the pathogenesis of the encountered events, because they are a clinically significant problem in RA patients receiving TNF-α-blocking therapy.

## Abbreviations

CI = confidence interval; DAS28 = disease activity score including 28-joint counts; DMARD = disease-modifying antirheumatic drug; ELISA = enzyme-linked immunosorbent assay; pt-yr = patient-year; RA = rheumatoid arthritis; Th1/Th2 = T helper cell type 1/2; TNF = tumor necrosis factor.

## Competing interests

The author(s) declare that they have no competing interests.

## Authors' contributions

MF participated in the study design, carried out the data collection and statistical analysis, and drafted the manuscript. WV participated in the study design, carried out the data collection, and helped to write the manuscript. MC participated in the study design and coordination and helped in the writing and revision of manuscript. EdJ participated in the study design and the data collection and helped to write the manuscript. PvdK and PvR helped to write and critically revise the manuscript and gave final approval of the manuscript. MF and WV contributed equally to the article. All authors read and approved the final manuscript.

## Figures and Tables

**Table 1:** Baseline characteristics of patients with rheumatoid arthritis (RA) studied

	Given TNF-α-blocking therapy		Controls^a^
		
Characteristic	All patients *N *= 289	Patients with dermatological events *N *= 72	*N *= 289
Male sex, no. (%)	89 (31)	20 (28)	110 (38)
Age (yr) at diagnosis, mean (SD)	44.5 (14.7)	43.4 (12.7)	54.6 (14.1)**
RF-positive, no. (%)	249 (87)	68 (94)	205 (71)*
Disease duration (yr) at baseline, median (range)	9.2 (0.1–44.9)	10.3 (0.3–44.9)^†^	6.2 (0.0–12.6)**
DAS28 at baseline, mean (SD)	5.9 (1.1)	6.1 (1.1)	3.6 (1.4)**
ANA-positive at baseline, no. (%)^b^	112 (50)	33 (49)	118 (41)
Prior DMARDs, median (range)	4 (1–10)	5 (2–8)	1 (0–6)**
Prednisolone at baseline, no. (%)	112 (39)	34 (47)	21 (7)**

^a^Not given TNF-α-blocking therapy. ^b^ANA at start was present in respectively 261 and 67 patients on TNF-α-blocking therapy. *P < 0.001, **P < 0.0001, compared with RA patients on TNF-α-blocking therapy; ^†^*P *< 0.001 compared with RA patients on TNF-α-blocking therapy who experienced no dermatological events. ANA, antinuclear antibody; DAS28, disease activity score based on 28 joints; DMARD, disease-modifying antirheumatic drug; RF, rheumatoid factor; SD, standard deviation; TNF, tumor necrosis factor.

**Table 2:** Dermatological events in patients with rheumatoid arthritis (RA) given TNF-α-blocking therapy

Nature of event	Events	Time to event (months)		Events during treatment	Major events	Histology	DMARDs^b^	Prednisolone^b^	Permanent withdrawal of anti-TNF-α^c^
	
	No. (%)	Median^a^	Range	No. (%)	No. (%)	No. (%)	No. (%)	No. (%)	No. (%)
Infection	33 (25.8)	9.1	1.1–61.1	24 (73)	0	5 (15)	20 (61)	21 (64)	4 (12)
Eczema	20 (15.6)	7.1	0.2–49.9	16 (80)	1 (5)	4 (20)	8 (40)	7 (35)	3 (15)
Drug-related eruption	15 (11.7)	1.9	0.1–18.8	15 (100)	1 (7)	12 (80)	6 (40)	6 (40)	7 47)
Ulcers	9 (7.0)	13.6	0.3–52.5	3 (33)	1 (11)	2 (22)	7 (78)	4 (44)	1 (11)
Skin tumor, benign	7 (5.5)	12.9	2.0–18.1	7 (100)	0	2 (29)	5 (71)	4 (57)	0
Skin tumor, malignant	5 (3.9)	4.5	1.1–38.0	4 (80)	0	5 (100)	2 (40)	2 (40)	1 (20)
Xerosis cutis	6 (4.7)	8.9	4.2–26.3	6 (100)	0	1 (16)	4 (67)	1 (17)	1 (17)
Vasculitis	5 (3.9)	12.0	1.5–49.9	4 (80)	0	4 (80)	3 (60)	5 (100)	1 (20)
Actinic keratosis	5 (3.9)	26.3	4.5–112.9	2 (40)	0	3 (60)	5 (100)	2 (40)	0
CVI/varices	4 (3.0)	24.0	1.7–33.6	3 (75)	0	0	3 (75)	2 (50)	0
Psoriasis/psoriasiform	3 (2.3)	15.5	8.4–50.1	3 (100)	0	3 (100)	0	2 (67)	1 (33)
Edema	3 (2.2)	8.2	4.0–39.6	2 (67)	0	1 (33)	1 (33)	1 (33)	0
Stasis dermatitis	3 (2.2)	17.5	14.6–42.1	3 (100)	0	1 (33)	1 (33)	1 (33)	0
Seborrheic dermatitis	2 (1.5)	0.4, 19.8	–	2 (100)	0	0	0	0	0
Other event	8 (6.0)	5.0	1.9–25.9	6 (75)	0	4 (50)	4 (50)	2 (25)	2 (25)
Total	128 (100)	9.1	0.1–112.9	100 (78)	3 (2)	47 (37)	69 (54)	60 (47)	21 (16)

^a^Median and range given for three cases or more; individual data given for two cases or fewer. ^b^Number of patients with concomitant DMARDs and prednisolone at the time of event. ^c^Permanent discontinuation of TNF-α-blocking therapy because of the event. DMARD, disease-modifying anti-rheumatic drug; TNF, tumor necrosis factor.

**Table 3:** Skin infections in patients with rheumatoid arthritis (RA) given TNF-α-blocking therapy

		Time to event							
								
Infection	No. of events	Median	Range	Drug^a ^(no.)	Active treatment^b ^(no.)	Rechallenge (no.)	Permanent withdrawal of anti-TNF-α^c ^(no.)	Biopsy (no.)	Cultured species
***Fungal***	20	8.7	1.1–61.1						
Dermatomycosis	9			A 3, I 4, E 2	7		0	1	*Trichophyton verrucosum *(1) *T. rubrum *(1)
Onychomycosis	3			A 3	3		0	0	
Combination	5			A 3, I 1, L 1	4		0	1	*Trichophyton rubrum *(3) *T. mentagrofytes *(1)
Candidiasis	3			I 3	2		0	0	*Candida *spp. (2)
***Bacterial***	11	9.5	1.4–52.5						
Folliculitis	5			A 3, E 2	4	yes, negative	1	2	*Staphylococcus aureus *(1)
Erysipelas	3			E 2, I 1	3	yes, negative	2	1	
Bacterial superinfection of eczema	2			A 1, I 1	1	yes, positive	1	0	
Furuncle	1			I 1	0		0	0	
***Viral – herpes zoster***	2	17.3, 40.9^d^		A 1, I 1	0		0	0	

^a^A, adalimumab; I, infliximab; E, etanercept; L, lenercept. ^b^During active treatment with TNF-α-blocking therapy. ^c^Permanent discontinuation of TNF-α-blocking therapy due to the event.^d ^Individual values

**Table 4:** Drug-related skin eruptions in patients with rheumatoid arthritis (RA) given TNF-α-blocking therapy

Patient no.	Age (yr)	Sex	Drug^a^	Route	Type of eruption	Clinical description	Localization	Time to event (mo)	Biopsy	Comedication^b^	Therapy	Rechallenge	Permanent withdrawal of anti-TNF-α	Course
1	62	f	A	i.v.	Eczematous	Erythematosquamous plaques and papules	Neck/ axillary/ legs	4.5	Yes	naproxen	Local	positive	No	Recurring
2	71	m	A	i.v.	Exanthematous lichenoid	Maculopapular exanthema	Generalized	0.7	Yes	prednisolone, naproxen, paracetamol	Local	positive	Yes	Recovery
3	77	m	E	s.c.	Exanthematous	Macular exanthema	Generalized	6.8	Yes	prednisolone, naproxen, omeprazole	Local	positive	No	Recurring
4	67	m	E	s.c.	Lichenoid	Macular exanthema, purpura	Generalized	1.5	Yes	diclofenac, omeprazole, triamterene, furosemide, candesartan	Topical, systemic	No	Yes	Recovery
5	69	f	I	i.v.	Eczematous	Erythematous plaque	Right cheek	0.1	Yes	MTX, pantoprazole, atenolol, calcium, hydrochlorothiazide	Topical	positive	No	Recurring
6	88	f	I	i.v.	Eczematous urticarial	Erythematosquamous macula, purpura	Lower arms/legs	3.9	Yes	leflunomide, carbasalate calcium, omeprazole, furosemide, simvastatin, paracetamol	Topical	No	Yes	Recovery
7	68	f	I	i.v.	Eczematous urticarial	Erythematosquamous plaques, urticaria, excoriations, lichenification, purpura	Generalized	10.3	No	AZA, furosemide, oxazepam, enalapril, spironolactone, metoprolol, flixotide, formoterol	Topical, systemic	negative	No	Recovery
8	60	f	I	i.v.	Exanthematous	Stippled exanthema	Generalized	0.5	Yes	naproxen, omeprazole	Topical	No	Yes	Recovery
9	53	f	I	i.v.	Exanthematous	Exanthema	Upper arms/legs	0.2	No	indomethacin	Topical	positive	No	Recurring
10	73	f	I	i.v.	Exanthematous, with purpura	Exanthema, purpura	Lower legs	18.8	No	MTX, folic acid, prednisolone, morphine, loperamide, latanoprost	Topical	No	Yes	Recovery
11	70	f	I	i.v.	Exanthematous urticarial	Exanthema, urticaria	Arms/ trunk	16.6	Yes	leflunomide	None	positive	No	Recurring
12	35	f	I	i.v.	Exanthematous urticarial, with purpura	Macular exanthema, uricaria, purpura	Trunk/ axillary/ groins	1.9	Yes	none	Topical	-	Yes	Recovery
13	58	f	I	i.v.	Lichenoid	Erythema, hyperpigmentation, atrophy	Upper legs	15.5	Yes	leflunomide, meloxicam, metoclopramide, acenocoumarol, digoxin	None	No	Yes	Recovery
14	58	f	L	i.v.	Exanthematous	Papular exanthema	Generalized	0.4	Yes	none	Topical	positive	No	Recurring
15	68	m	L	i.v.	Exanthematous lichenoid	Maculopapular exanthema	Generalized	1.7	Yes	prednisolone, paracetamol	Topical	negative	No	Recovery

Events numbers 5 and 11 occurred in the same patient, as did events numbers 2, 3, and 15. ^a^A, adalimumab; Age = age ar event; I, infliximab; E, etanercept; L, lenercept. ^b^MTX, methotrexate; AZA, azathioprine. f, female; i.v., intravenous; m, male; s.c., subcutaneous.

**Table 5:** Other dermatological events in patients with rheumatoid arthritis (RA) given TNF-α-blocking therapy

Patient no.	Age (yr)	Sex	Diagnosis	Drug^a^	Active treatment	Event	Clinical description	Localization	Time to event	Biopsy	Comedication^b^	Permanent withdrawal anti-TNF-α	Therapy	Course
1	56	f	RA	A	Yes	Lymphomatoid papulosis-like eruption	Macular erythematosquamous lesions	Lower arms, upper legs and trunk	2.6	Yes	naproxen	No	None	Recovery
2	53	f	RA	A	Yes	Rosacea	Diffuse erythema, scaling, telangiectasias	Head and face	1.9	Yes	prednisolone, captopril, indomethacin, midazolam	No	Topical	Persisting
3	74	f	RA	E	Yes	Pruritus	Itch	Trunk	2.5	No	None	No	Topical	Unknown
4	61	f	RA	I	No	Ecchymoses	Ecchymoses	Hands and feet	25.9	No	AZA, prednisolone	No	Topical	Partial recovery
5	58	f	RA	I	Yes	Drug-induced systemic lupus emythematosus	Discoid erythematous lesions, aphthous lesions, ANA positive, anti-ds-DNA positive	Hands, face, scalp	20.0	No	MTX	Yes	Topical and systemic	Recovery, no rechallenge
6	68	m	RA	I	Yes	Transient swelling of unknown cause	Transient swelling 2 × 3 cm	Scalp	20.0	No	MTX, folic acid, naproxen	No	None	Recovery
7	52	f	RA	L	Yes	Dermatomyositis	Livid erythema, raised CPK, decreased proximal muscular strength	Inner upper arms and legs	2.5	Yes	None	Yes	None	Recovery
8	53	m	RA	L	No	Erythema nodosum	Painful erythematous nodules	Lower legs	7.4	Yes	AZA, naproxen, paracetamol	No	Topical	Partial recovery

^a^A, adalimumab; I, infliximab; E, etanercept; L, lenercept. ^b^MTX, methotrexate; AZA, azathioprine. CPK, creatinine phosphokinase; f, female, m, male.
